# A single institution retrospective analysis on survival based on treatment paradigms for patients with anaplastic oligodendroglioma

**DOI:** 10.1007/s11060-021-03781-z

**Published:** 2021-06-14

**Authors:** Nancy Ann Oberheim Bush, Jacob S. Young, Yalan Zhang, Cecilia L. Dalle Ore, Annette M. Molinaro, Jennie Taylor, Jennifer Clarke, Michael Prados, Steve E. Braunstein, David R. Raleigh, Susan M. Chang, Mitchel S. Berger, Nicholas A. Butowski

**Affiliations:** 1 Division of Neuro-Oncology, Department of Neurological Surgery, University of California, San Francisco, CA USA; 2 Department of Neurology, University of California, San Francisco, CA USA; 3 Department of Neurological Surgery, University of California, San Francisco, CA USA; 4 Department of Radiation Oncology, University of California, San Francisco, CA USA

**Keywords:** Glioma, Anaplastic Oligodendroglioma, Chemotherapy, Radiation

## Abstract

**Introduction:**

Anaplastic oligodendrogliomas are high-grade gliomas defined molecularly by 1p19q co-deletion. There is no curative therapy, and standard of care includes surgical resection followed by radiation and chemotherapy. However, the benefit of up-front radiation with chemotherapy compared to chemotherapy alone has not been demonstrated in a randomized control trial. Given the potential long-term consequences of radiation therapy, such as cognitive impairment, arteriopathy, endocrinopathy, and hearing/visual impairment, there is an effort to balance longevity with radiation toxicity.

**Methods:**

We performed a retrospective single institution analysis of survival of patients with anaplastic oligodendroglioma over 20 years.

**Results:**

159 patients were identified as diagnosed with an anaplastic oligodendroglioma between 1996 and 2016. Of those, 40 patients were found to have AO at original diagnosis and had documented 1p19q co-deletion with a median of 7.1 years of follow-up (range: 0.6–16.7 years). After surgery, 45 % of patients were treated with radiation and chemotherapy at diagnosis, and 50 % were treated with adjuvant chemotherapy alone. The group treated with chemotherapy alone had a trend of receiving more cycles of chemotherapy than patients treated with radiation and chemotherapy upfront (p = 0.051). Median overall survival has not yet been reached. The related risk of progression in the upfront, adjuvant chemotherapy only group was almost 5-fold higher than the patients who received radiation and chemotherapy (hazard ratio = 4.85 (1.74–13.49), p = 0.002). However, there was no significant difference in overall survival in patients treated with upfront chemotherapy compared to patients treated upfront with chemotherapy and radiation (p = 0.8). Univariate analysis of age, KPS, extent of resection, or upfront versus delayed radiation was not associated with improved survival.

**Conclusions:**

Initial treatment with adjuvant chemotherapy alone, rather than radiation and chemotherapy, may be an option for some patients with anaplastic oligodendroglioma, as it is associated with similar overall survival despite shorter progression free survival.

## Introduction

Anaplastic oligodendrogliomas (AO) are grade III tumors which harbor the 1p/19q co-deletion, and are known to be chemosensitive [1, 2]. Current standard of care treatment is surgical resection followed by radiation therapy (RT) and chemotherapy, although patients with poor performance status may only receive a subset of these modalities or palliative oriented treatment. Small case series originally demonstrated a benefit for patients with recurrent tumors who were treated with procarbazine, lomustine, and vincristine (PCV) [1, 2]. These early reports led to two randomized trials to clarify the role of chemotherapy for patients with AO at the time of initial diagnosis: Radiation Therapy Oncology Group (RTOG) 9402 and European Organization for Research and Treatment of Cancer (EORTC) 26,951. In RTOG 9402, PCV given before RT doubled the median survival compared to RT alone for patients with co-deleted tumors (14.7 years vs. 7.3 years) [3]. Moreover, in EORTC 26,951, patients with anaplastic tumors who received six cycles of PCV after 59.4 Gy of RT had improved survival compared to patients who received RT alone (42.3 vs. 30.6 months), and there was a strong trend for additional benefit in patients harboring the 1p/19q co-deletion [4]. In a population based analysis of nearly 15,000 patients, postoperative radiotherapy did not improve survival for AO, although it did improve survival for glioblastoma and anaplastic astrocytoma [5]. Moreover, long term analysis of the NOA-04 randomized control trial found no difference in survival for anaplastic tumors initially treated with chemotherapy alone compared to radiation therapy alone [6]. To help determine the optimal upfront treatment, the international phase III trial, “CODEL“ randomized 36 patients with 1p19q co-deleted tumors to RT with concurrent and adjuvant temozolomide (TMZ) (12 patients) compared to radiation alone (12 patients) or TMZ alone (12 patients). Initial analysis with a median follow-up of 7.5 years showed that 83.3 % of patients treated with TMZ alone had progressed compared to 37.5 % of the patients treated on the radiation therapy arms, and PFS was significantly shorter in TMZ alone treated patients (p = 0.014) [7]. However, overall survival did not significantly differ between the arms in patients with the IDH mutation (p = 0.2) in this underpowered study. As such it remains unclear if chemotherapy only as upfront treatment for patients with anaplastic oligodendrogliomas is an inferior choice taking consideration that for many patients living over 10 years, any possible long term effects of radiation, such as cognitive impairment, arteriopathy, endocrinopathy, and hearing/visual impairment, could lead to major disability and significantly impair patient’s quality of life [8, 9], although the magnitude of any long term cognitive sequelae of radiation is unknown and may be improved by contemporary radiation techniques [10]. As such, the optimal initial treatment that balances longevity with quality of life has yet to be determined.

## Methods

This study was approved by the University of California, San Francisco IRB. Between 1996 and 2016, patients with histologically proven AO at UCSF were identified. A diagnosis of anaplastic oligodendroglioma was made by the pathologist when there was confirmation of a 1p/19q codeletion and either an elevated MIB index or the presence of anaplastic features, such as pleomorphic nuclei, hypercellularity, microvascular proliferation, or necrosis. Of these patients, those that did not follow-up at UCSF after surgery and cases where 1p19q co-deletion was either not tested or found to be negative were not included in the analysis. Only patients with AO at time of initial diagnosis were included and patients who progressed from Grade II oligodendrogliomas were not included. Demographic data, performance status, location, extent of resection (biopsy, subtotal resection or gross total resection), treatment modalities and pathology, including 1p19q co-deletion florescence in situ hybridization (FISH) testing were collected from the medical record. Patients were categorized as either receiving RT alone, RT plus chemotherapy (TMZ), chemotherapy alone (TMZ), or refused treatment. Pre-operative and post-operative tumor volumes were quantified by using BrainLab Smartbrush software (Brainlab, Munich, Germany). Manual segmentation of tumor volume was performed with region-of-interest analysis based on T1 post-gadolinium sequences and the T2 FLAIR sequences from pre- and post-operative MRI scans, respectively. EOR was calculated as: (pre-operative tumor volume – post-operative tumor volume)/pre-operative tumor volume x 100 %. Overall survival and progression free survival were assessed from time of initial diagnosis to time of death or last follow-up with Kaplan-Meier curves using the statistical software R (http://www.r-project.org/). Hazard ratios with corresponding 95 % confidence intervals (CIs) for overall survival (OS) and progression free survival (PFS) were calculated. Data collection was closed on October 10th 2019. Patients received serial surveillance MRI scans and clinical examinations approximately every 2–6 months.

## Results

Between the years of 1996–2016, 159 cases of histologically proven AO were identified. Of these, 57 patients were deemed to have AO at original diagnosis, did not progress from a lower grade tumor, and were treated at UCSF, thus allowing for appropriate clinical follow-up. Of these patients, 40 had documented 1p19q co-deletion and thus were included in the analysis (30 of these patients were also tested for the presence of IDH mutation either by IHC or genomic analysis and all were positive for either an IDH1 or 2 mutation; in other cases IDH testing was not performed as it was not routinely done when this study commenced). The remaining patients did not have sufficient tissue available for 1p19q testing. The median age at diagnosis was 44 (range: 22.7–83.9 years), and there was male predominance with 63 % male and 37 % female (Table 1). Overall the patients had excellent KPS (mean 90, range 70–100) at diagnosis. 48 % of patients had left hemisphere tumors, 40 % right hemisphere and 12 % were bilateral. 68 % were located in the frontal lobe. No tumors were multifocal in nature.

**Table 1:** Demographics

	1p19q co-deleted tumors (N = 40)	XRT/TMZ (n = 18)	TMZ (n = 20)
*Gender *			
F	15 (37.5 %)	4 (22 %)	9 (45 %)
M	25 (62.5 %)	14 (78 %)	11 (55 %)
* Age at diagnosis*			
(18,30]	4 (10.0 %)	0	4 (20 %)
(30,50]	26 (65.0 %)	12 (67 %)	12 (60 %)
(50,70]	9 (22.5 %)	6 (33 %)	4 (20 %)
(70,100]	1 (2.5 %)	0	0
* KPS *			
Mean (SD)	87.8 (6.2)	86.1	89
Median	90	90	90
Q1, Q3	90.0, 90.0	90.0, 90.0	90.0, 90.0
Range	70.0–100.0	70.0–100.0	80.0–90.0
*Tumor hemisphere*			
Bilateral	5 (12.5 %)	1 (6 %)	4 (20 %)
Left	19 (47.5 %)	11 (61 %)	7 (35 %)
Right	16 (40.0 %)	6 (33 %)	9 (45 %)
* Tumor location*			
Frontal	27 (67.5 %)	13 (72 %)	12 (60 %)
Parietal	8 (20 %)	3 (17 %)	6 (30 %)
Temporal	3 (7.5 %)	1 (6 %)	2 (10 %)
Occipital	2 (5.0 %)	1 (6 %)	0
*Tumor volumes*^a^			
T1 post gad EOR (SD)	94.1 % (19.7 %)	87.5 % (30.0 %)	98.4 % (5.6 %)
FLAIR EOR (SD)	69.7 % (34.9 %)	72.2 % (33.1 %)	63.7 % (38.7 %)
T1 post gad pre op volume (SD)	19.1 cm^3^ (30.6 cm^3^)	23.7 cm^3^ (36.8 cm^3^)	11.6 cm^3^ (18.6 cm^3^)
FLAIR pre op volume (SD)	93.0 cm^3^ (66.4 cm^3^)	97.4 cm^3^ (78.4 cm^3^)	90.2 cm^3^ (57.8 cm^3^)
T1 post gad residual volume (SD)	0.7 cm^3^ (2.1 cm^3^)	1.3 cm^3^ (2.6 cm^3^)	0.4 cm^3^ (1.7 cm^3^)
FLAIR residual volume (SD)	32.7 cm^3^ (43.1 cm^3^)	28.9 cm^3^ (35.3 cm^3^)	41.1 cm^3^ (51.6 cm^3^)
Number of craniotomies	1.6	1.4	1.8

^a^T1 post gad volume and EOR was not calculated for patients with nonenhancing tumors (n = 7)

**Fig. 1:**
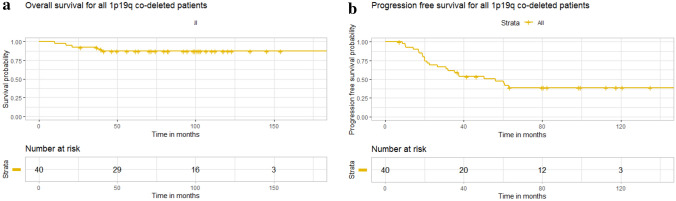
Kaplan–Meier curve for overall survival and progression free survival

**Fig. 2:**
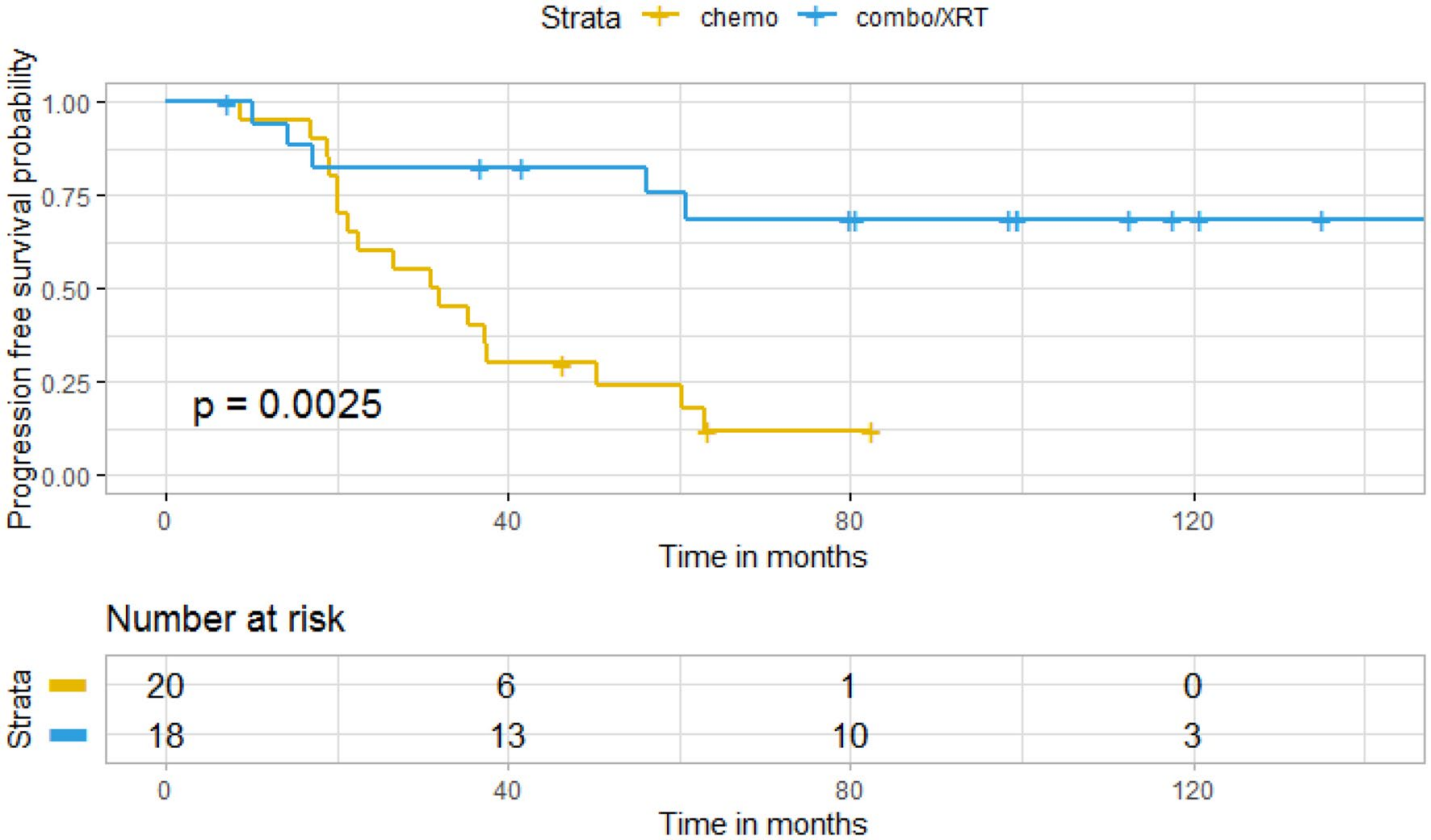
Kaplan–Meier curve for progression free survival by treatment group

Of the 40 patients included in the analysis, there was median follow-up of 7.1 years and 34 (85 %) were alive at the time of data collection. Median progression free survival (mPFS) was 56.3 months (95 % CI: 32.1 months-NA) and median overall survival (mOS) was not yet reached (Fig. 1). Of these patients 18 patients were treated with a combination of standard dose radiation and TMZ and 20 patients were treated with TMZ alone. Due to institutional preference, no patients were treated with PCV or CCNU. One patient was treated with radiation alone and another with no treatment and were therefore not included in the analysis of progression free and overall survival by treatment modality. Of the 18 patients treated with a combination of radiation and TMZ, all patients received TMZ during radiation therapy and 13 patients also received adjuvant TMZ. No patients received radiation therapy directly after the completion of temozolomide. The average number of adjuvant cycles was in this cohort was 9 (range 1–18). For patients treated with TMZ alone, the average number of cycles was 13.2 (range 6–40), which approached significance when compared to combination therapy (p = 0.051). One patient in this cohort remained on treatment with TMZ at the time of data censoring. Patients treated with radiation and TMZ had significantly longer PFS than patients treated with chemotherapy alone as monotherapy (157.8 months vs. 31.5 months, p = 0.0025) (Fig. 2). Univariate analysis revealed the related risk of progression for patients treated with chemotherapy alone at initial diagnosis was almost 5-fold higher than patients treated with a combination of radiation and TMZ (HR = 4.85 (1.74–13.49), p = 0.002). At the time of first progression, 65 % of patients received XRT and chemotherapy with the remaining patients either receiving additional chemotherapy alone or surgical intervention.

Residual FLAIR volume was also a significant predictor of progression free survival (HR 1.01, CI 1.00–1.02, p = 0.036). 17.5 % of cases had no contrast enhancement, and 32.5 % of patients had very minimal or wispy enhancement. Accordingly, the extent of resection of contrast enhancing disease, when present, was 94.1 %, although this was not significant (HR 1.02, CI 0.97–1.07, p = 0.383). There was no association with residual FLAIR or enhancing disease or EOR and overall survival.

**Fig. 3:**
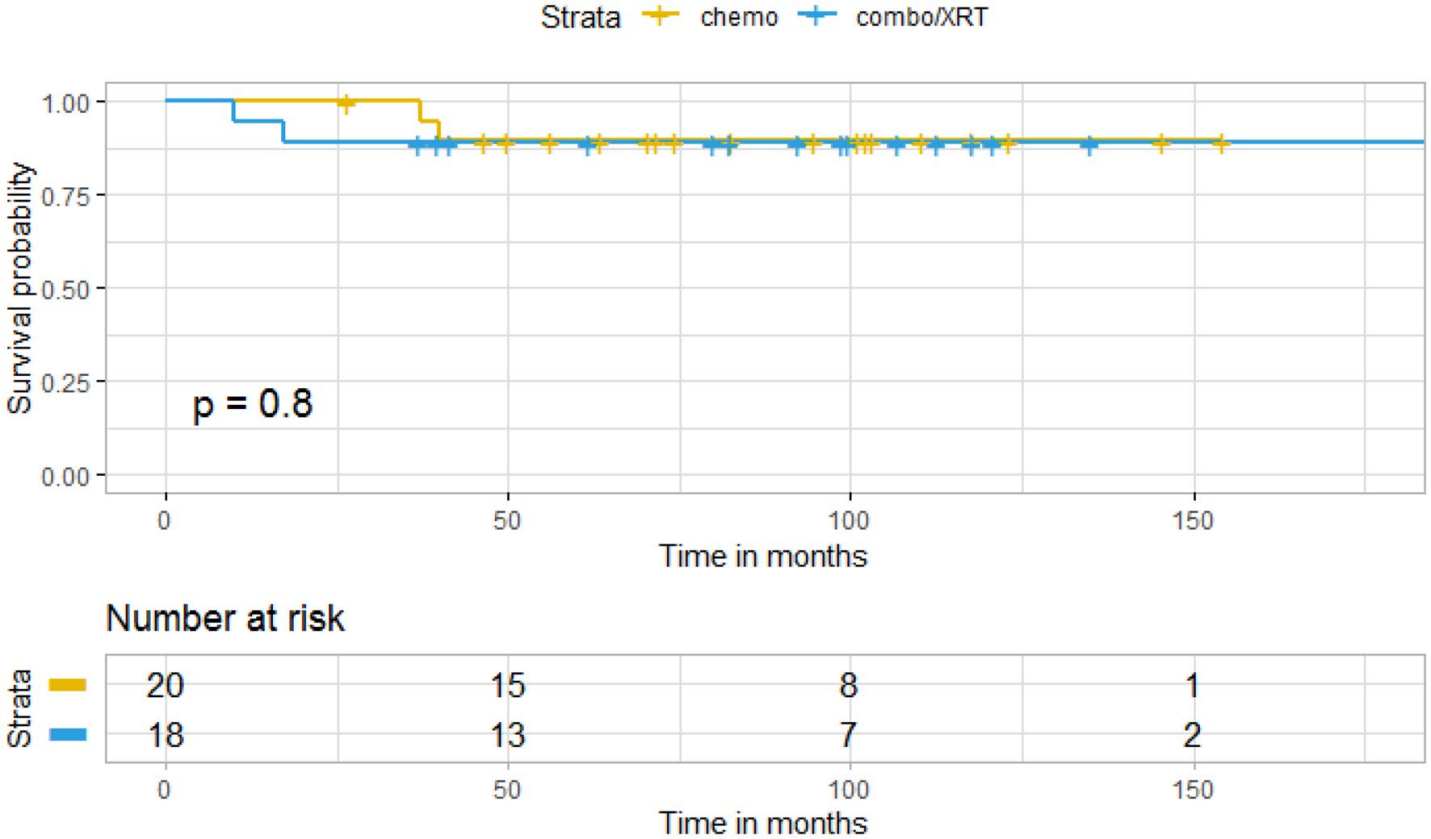
Kaplan–Meier curve for overall survival by treatment group

**Fig. 4:**
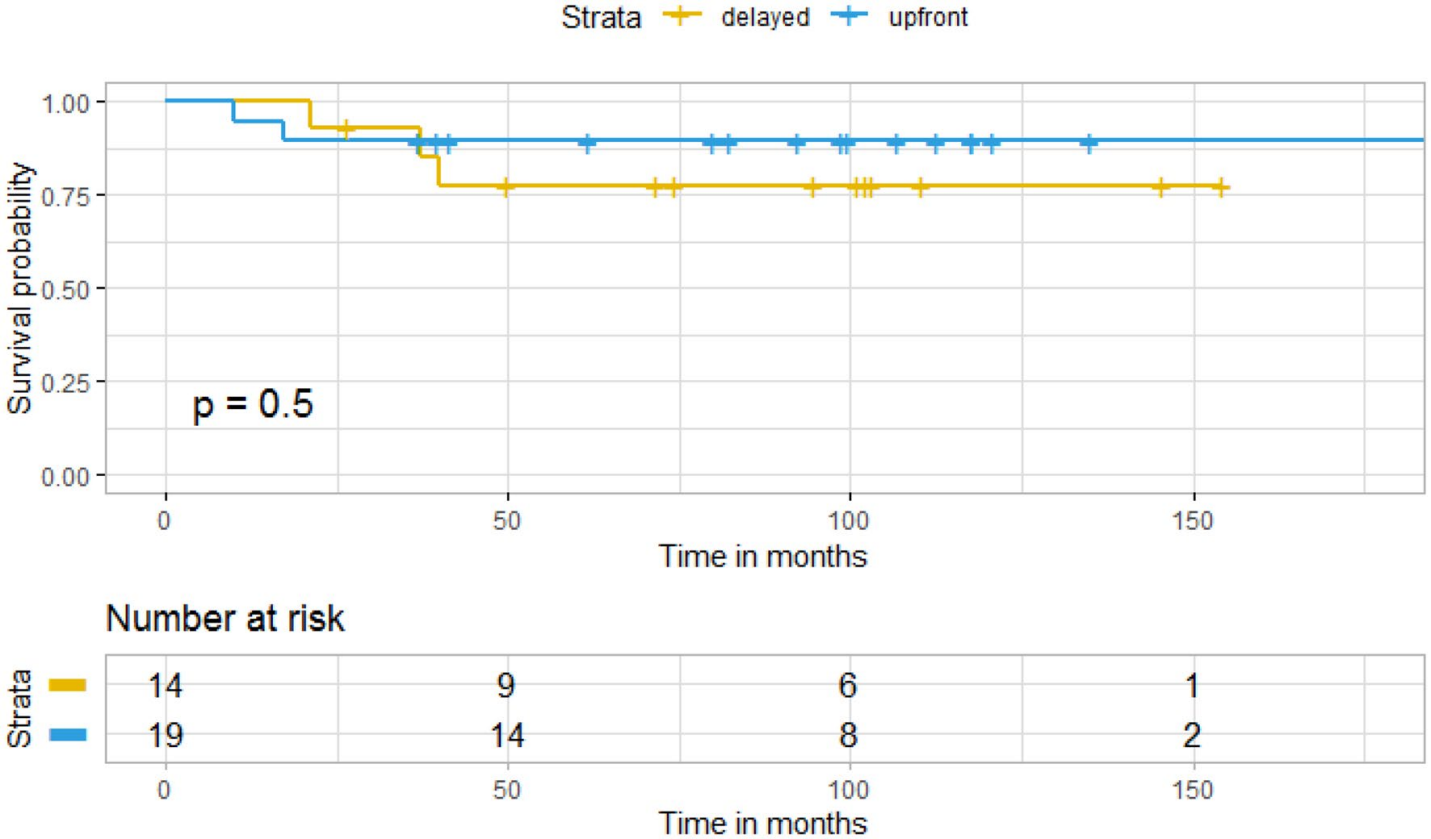
Kaplan–Meier curve for overall survival in upfront versus delayed radiation

There was no significant difference in OS in patients who were treated with TMZ upfront compared to patients treated with TMZ and radiation at initial diagnosis (p = 0.8) (Fig. 3). Of the 20 patients who received chemotherapy alone at initial diagnosis, 14 received radiation at the time of progression (median 31.5 months). There was no significant difference in OS in patients who received radiation at initial diagnosis compared to those who received radiation at time of progression (p = 0.5) (Fig. 4). Univariate analysis of age, KPS and extent of resection did not reveal any variables significantly associated with overall survival (Table 2).

**Table 2:** Univariable Overall Survival analysis for 1p19q co-deleted cases

Variables	HR (univariable)
Gender	
M	–
F	2.35 (0.39–14.07, p = 0.349)
Age at diagnosis	
(0,44]	–
(44,100]	0.70 (0.12–4.20, p = 0.697)
KPS > 90	
1	–
0	1.19 (0.13–10.69, p = 0.874)
GTR vs. biopsy/STR	
Biopsy/STR	–
GTR	1.44 (0.24–8.61, p = 0.691)
Chemo vs. XRT or combo	
Combo/XRT	–
Chemo	0.83 (0.12–5.88, p = 0.850)
Upfront vs. Delayed XRT	
Upfront	–
Delayed	1.96 (0.33–11.76, p = 0.459)
T1 post gad EOR	
94.1	1.02 (0.97–1.07, p = 0.383)
T2 FLAIR EOR	
69.7	0.99 (0.98–1.00, p = 0.116)
T1 post gad volume	
0.7	0.96 (0.75–1.123, p = 0.759)
T2 FLAIR post op volume	
32.7	1.01 (1.00–1.02, p = 0.036)

## Discussion

Here we report extended survival for anaplastic oligodendrogliomas patients, which in the past have been thought to have poor prognosis. As with other low and high grade gliomas, minimizing residual T2 FLAIR disease was associated with increased progression free survival [11, 12]. We found that patients treated with radiation and TMZ upfront have a significantly longer progression free survival compared to patients treated with TMZ at initial diagnosis. However, strikingly, there was no difference in overall survival in patients who have delayed radiation treatment to time of progression. On average radiation was delayed 2.6 years. Of note, the patients who received TMZ monotherapy at diagnosis did have a trend to have more cycles of TMZ compared to patients also treated with radiation, although this did not reach significance (p = 0.051).

One limitation of this study, similar to prior published reports, is its relatively small sample size, and more advanced multivariate analysis could not be performed. However, we restricted the analysis to patients with confirmed 1p19q co-deletion as most prior studies have looked at a heterogeneous population of either all anaplastic tumors, or a combination of anaplastic oligodendroglioma with anaplastic oligoastrocytoma which may significantly impact overall survival. Additionally, the study looks specifically at TMZ mainly due to institutional preference and therefore it remains unclear if these results will translate to other chemotherapy regimes including CCNU and PCV. Furthermore, this is a retrospective study, and there may have been some selection bias in the patients who did and did not receive radiation therapy. For example, there was a higher number of patients with bilateral tumors in the upfront chemotherapy group which may represent preference to limit radiation in cases that would require a large radiation field given the concern for cognitive decline. Finally, as our practice does not to routinely assess executive function or cognitive processing speed, we are unable to directly evaluate for cognitive changes between the two cohorts, which remains an important area of future investigation.

Given the responsiveness of these co-deleted tumors to chemotherapy, some reports have questioned the benefit that surgical extent of resection has on overall survival and malignant transformation for patients with oligodendroglial tumors [13, 14]. Nevertheless, surgery, radiation and chemotherapy remains mainstay in the upfront treatment of anaplastic oligodendrogliomas [15]. Here, despite the relatively small sample size, we found an association between EOR of the T2 FLAIR disease and progression free survival. Several studies have suggested that chemotherapy without upfront radiation may be a viable option for patients with grade II oligodendrogliomas [16–18]; this also may hold true for patients diagnosed with anaplastic oligodendroglioma [19]. One major benefit of prolonging the use of radiation in patients would be limiting the potential negative long term effects of radiation, including potential cognitive [7, 9] and some emerging evidence suggests it may alter wide scale functional connectivity [20]. In our study, the median age of diagnosis was 44, and the median overall survival was not reached at greater than 10 years of follow-up. As such delaying radiation in young patients who have long survival rates may limit the cognitive changes they experience over time and significantly impact their quality of life [21–23]. Additionally, patients with brain radiation are at risk for radiation induced vasculopathy, endocrinopathies and secondary malignancies [24]. All of these risks may be decreased with delay of radiation treatment. However, our study demonstrated that radiation was delayed on average 2.6 years, and whether this delay would have a meaningful impact on the potential long term sequala of radiation remains unknown. Radiation therapy is also known to lead to radiographic changes that may be misinterpreted as tumor progression, and as such delaying radiation may improve surveillance imaging interpretation. Radiation therapy also may render re-operation more risky due to changes in skin turgor and wound healing, and as such delaying radiation may have the added benefit of improving the safety of re-resection [25, 26]. Delaying radiation therapy and treating with chemotherapy upfront may allow for earlier disease progression, but may not influence overall survival, and therefore may be a viable treatment option in patients who are concerned with the potential long term effects of radiation.

Alternatively, upfront radiation may result in fewer cycles of chemotherapy and prolong progression free survival, two potentially desirable outcomes for patients. A reduction in the number of chemotherapy cycles could improve patient quality of life, minimize treatment related toxicities, and reduce costs. While these observations need to be investigated further in randomized trials, we believe this data warrants a discussion with patients regarding the utility of early radiation therapy versus the potential side-effects.
